# Clinical characteristics of infantile haemangioma in twins: a retrospective study

**DOI:** 10.1186/s12887-024-04602-8

**Published:** 2024-02-13

**Authors:** Zhengwei Sun, Miaomiao Li, Changxian Dong, Shiwei Mei

**Affiliations:** 1 Department of Interventional Radiology and Hemangioma, Guangdong Province Woman and Children Hospital, Guangzhou, People’s Republic of China; 2 Department of Hemangioma and Vascular Malformation, Henan Provincial People’s Hospital, Zhengzhou, People’s Republic of China

**Keywords:** Infantile hemangioma, Twins, Clinical characteristics, Hypoxia-inducible factor, Disease perceptions

## Abstract

**Background:**

Infantile hemangioma is one of the most common benign soft tissue tumors in infants. The pathogenesis of infantile hemangioma remains unclear and twin studies regarding its incidence may help clarify disease pathogenesis. Thus, this study aimed to analyze the clinical characteristics of infantile hemangioma in twin patients and discuss its clinical incidence.

**Methods:**

We retrospectively analyzed the data of 83 pairs of twins with infantile hemangioma admitted to the Guangdong Provincial Women and Children Hospital and Henan Provincial People’s Hospital between May 2016 and May 2022. Thirty-one pairs of twins among whom both developed infantile hemangioma and 52 pairs of twins among whom only one twin was affected were included. Analysis was performed using the Spearman correlation. Additionally, we analyzed the influence of factors such as sex, twin zygosity, preterm birth, birth weight, and assisted reproduction on the clinical characteristics of twins.

**Results:**

We observed that disease occurrence in both twins correlated with assisted reproduction (χ2 = 13. 102, *P* < 0.05) and preterm birth (χ2 = 36.523, *P* < 0.05). Twin zygosity (χ2 = 0.716, *P* > 0.05) and total birth weight of twins (t=-3.369, *P* > 0.05) were not correlated with infantile hemangioma. However, among twins, the ones with lesser birth weight were more likely to develop infantile hemangioma.

**Conclusions:**

The clinical characteristics of infantile hemangioma in twins were consistent with their epidemiological characteristics. Female sex, preterm birth, less birth weight, and assisted reproduction increased the probability of morbidity in both twins. Analysis of the characteristics of infantile hemangioma in twins may assist further research and clinical treatment.

## Background

Infantile hemangioma (IH) is one of the most common benign soft tissue tumors during infancy. Infants born prematurely and with low birth weight have a higher incidence rate, approximately 4–5% of newborns are affected, and the male to female ratio is approximately 1:3 [1–3].In most cases, IH is typically small and may regresses naturally, but larger and faster-growing IHs may leave permanent pigmentation, vascular dilatation, fibrofatty tissue buildup, and scarring following regression. Moreover, IH in specific sites can lead to major problems such as disfigure, breathing difficulties, visual impairment, restricted joint movement, congestive heart failure and even death [1, 4, 5]. The incidence of IH appears to have increased significantly over the last 40 years, but the pathogenesis of IH remains unclear [6, 7].

Morbidities in twins are often observed during clinical diagnosis and treatment [8]. Previous studies identified twin pregnancy as a risk factor for IH [5, 9]. Additionally, a multicenter prospective cohort study reported the incidence of hemangiomas in twins [10]. The study reported that factors affecting the incidence of hemangioma in twin infants included birth weight, sex, and gestational age. But there were several studies focus on characteristics of IH in twins and how the difference is generated.

Monozygotic twins share similar DNA sequences; however, they do not manifest diseases in the same way. Alternatively, dizygotic twins can provide a more distinct comparison [11]. Therefore, twin studies regarding the incidence of IH are significant for identifying disease pathogenesis due to their uniqueness. Herein, we analyzed the incidence of IH in twins treated in two centers and compared the clinical characteristics to explore the regularity of the incidence.

## Materials

### Patients

This study included twins with IH treated in the Department of Hemangioma at Guangdong Provincial Women and Children Hospital and the Department of Hemangioma at Henan Provincial People’s Hospital between May 2016 and May 2022. Patients were included if either twin was diagnosed with IH based on appearance, rupture or deformation, and color Doppler ultrasonography and magnetic resonance imaging findings. Also, no history of other diseases was reported. All children were followed up to the age of 2 years and their disease development and treatment responses were recorded. If only one twin developed IH, the other twin was followed up to the age of 2 years to ensure there were no hidden growth sites or missed lesions. During the follow-up, if the initially unaffected twin was found to be affected, they were included in the corresponding group.

A total of 83 pairs of twins were included in the study: 42 female twins, 33 male twins, and 8 pigeon pairs. Fifty-five and 28 pairs of twins were conceived via natural conception and artificial insemination, respectively. There were 70 pairs of monozygotic and 13 pairs of dizygotic twins. Thirty-one pairs of twins shared the disease, whereas, in 52 pairs, only one twin was affected by the disease. Table 1 details the characteristics of twins in the study.

**Table 1:** Characteristics of twins in the study

**Characteristics**	**One twin affected(** ***n*** ** = 52)**	**Both twins affected(** *n* ** = 31)**
Sex		
Male twins	29	4
Female twins	18	24
Pigeon pairs	5	3
Zygosity		
Monozygotic twins	42	28
Dizygotic twins	10	3
Assisted reproduction		
Artificial insemination	10	18
Natural conception	42	13
Preterm birth		
Preterm birth	6	24
Term birth	46	7
Sum of birth weight(kg)		
Median	5.706(IQR 5.611–5.801)	5.652(IQR 5.498–5.805)

Written informed consent was obtained from the parents or guardians to use the patients’ information. This study was approved by the Ethics Review Board of the institutions and was performed in accordance with the tenets of the Declaration of Helsinki.

### Data collection

Basic information about the twins, such as preterm birth and birth weight, and disease-related information, such as the number and location of IH, rupture or deformation, and complications, were collected through paper questionnaires, telephone inquiries, and face-to-face interviews. We also collected their prenatal ultrasound report and defined the twins’ zygosity based on whether they were reported to be monochorionic or dichorionic. Parents’ opinions—whether the twins looked the same—were also taken as references to define the twins’ zygosity. In addition, for mothers of twins, information regarding the method of conception and special circumstances surrounding the perinatal period were recorded. To ensure the standardization and accuracy of data collection and reduce expression bias in the inquiry process, most questions, except those regarding the gestational week and birth weight, were designed as yes/no questions.

### Statistical analysis

A χ2 test was used to identify variables for correlation analysis from data collected in the yes/no questionnaires and organized into crosstabs. In this study, the total sample size was 83 (*n* = 83), and no expected frequency was below 1 (t ≥ 1). Thus, we used the χ2 test with Yates’ correction if the expected frequency was < 5 in more than 20% of table cells; otherwise, we used Pearson’s chi-squared test. Additionally, we performed multivariate linear regression with backward elimination followed by a non-parametric test for other non-count data. All tests were performed using SPSS (IBM® SPSS Statistics, Version 26). The significance level was set at 5%.

## Results

### Sex

In the 83 pairs of twins, the rates of IH development in both twins and in only one twin were 37.3% (31/83) and 62.7% (52/83), respectively. There were 24 pairs of female twins, 4 pairs of male twins, and 3 pigeon pairs with both affected by IH. In 18 pairs of female twins, 29 pairs of male twins, and 5 pigeon pairs, only one twin was affected by IH. The outcome was χ2 = 14.269 (*P* < 0.05), suggesting sex and morbidity in both twins were correlated. Female twins were more likely to be both affected by IH.

### Zygosity

The 83 pairs of twins mainly consisted of monozygotic twins (70/83) and there were only 13 pairs of dizygotic twins. Among them, 28 pairs of monozygotic twins developed IH, while only one twin per 42 pairs of monozygotic twins developed IH. Additionally, in 3 pairs of dizygotic twins, both twins developed IH, while only one twin per 10 pairs of dizygotic twins developed IH. The outcome was χ2 = 0.716 (*P* > 0.05), suggesting no correlation between zygosity and morbidity.

### Assisted reproduction

Nearly two-thirds of the 83 pairs of twins (55/83) were natural conception, and the rest one-third pairs of twins (28/83) were conceived through artificial insemination using assisted reproductive technology and other related technologies. Among those who were conceived using assisted reproductive technology, 18 pairs of twins conceived through artificial insemination developed IH, while only one twin per the other 10 pairs developed IH. Additionally, both twins developed IH in 13 pairs of twins conceived through natural conception, while only one twin per the other 42 pairs developed IH. The outcome was χ2 = 13.102 (*P* < 0.05), suggesting that assisted reproduction and morbidity in both twins were correlated. Using assisted reproduction increased the probability of both twins affected by IH.

### Preterm birth

In the 83 pairs of twins, the rate of twins born preterm was 36.1% (30/83), and the rate of those born at term was 63.9% (53/83). Among the preterm twins, 24 pairs developed IH, while only one twin per the other 6 pairs developed IH. Additionally, 7 pairs of twins born at term developed IH, while only one twin per the other 46 pairs developed IH. The outcome was χ2 = 36.523 (*P* < 0.05), suggesting that preterm birth and morbidity in both twins were correlated. Preterm birth twins were more likely to be both affected by IH.

### Birth weight

Multivariate linear regression showed that there was no association between the summed birth weight of the twins and morbidity in both twins. Lower sum of birth weight didn’t increase the probability of both twins affected by IH.

However, for the 53 pairs of twins in which only one twin developed IH, 90.4% (47/53) of the affected twin had a lesser birth weight than the other one. The lower birth-weight one in twins was moer likely to be affected by IH.

## Discussion

IH is a common benign tumor in infants and young children; however, its pathogenesis is yet to be fully understood. Twins, particularly monozygotic twins can exhibit different disease manifestations although they share the same genetic information, which can provide a reference for understanding the pathogenesis of many diseases due to their unique biological characteristics. Therefore, studies on the incidence of IH in twins can provide meaningful data.

IH is believed to be derived from the embryo [12]. In this study, IH developed in twins of different sex and zygosity. Moreover, there was no obvious correlation with zygosity. This suggests that IH is a sporadic disease caused by abnormal embryonic development.

Female twins were more likely to both develop IH [13], whereas, in male twins, one twin was typically affected. Further, female twins in pigeon pairs were more likely to be affected. Twins conceived using assisted reproductive technology were more likely to develop morbidity. Preterm twins were more likely to both develop IH. Although the sum of twin birth weights did not appear to be associated with the development of other morbidities, lower birth weight twins were more likely to develop IH.

In a review of IH, IH incidence in female infants was 2.3–2.9 times that in male infants, and low birth weight and low gestational age were both significant risk factors [13, 14]. The data obtained in this study support these findings. The clinical characteristics of IH in twins were equivalent to the epidemiological characteristics.

The female sex is a recognized congenital factor for IH development [15]; however, the mechanism is not fully understood. The possibility of one affected twin in monozygotic female twins suggests that IH development can be attributed to embryonic development rather than genetic factors. Complex disease manifestations in twins can explain the differences in IH development in different embryos. Even if embryos are exposed to the same factors, the effects are probabilistic [16].

Increased morbidity in twins caused by assisted reproductive technology may be related to the use of estrogen and progesterone [17]. Estrogen is well-known for promoting the proliferation of hemangiomas. Further, increased estrogen levels in the maternal environment may impact the pathogenesis of hemangiomas [18]. Notably, the recent increase in the prevalence of IH may be related to the widespread application of assisted reproductive technology. Some studies have shown related trends but a specific link remains to be studied further [19].

Preterm birth and low birth weight are other known risk factors for IH. Some studies have suggested that preterm birth causes hemangioma growth through a hypoxia pathway because preterm infants are more prone to neonatal respiratory diseases than term infants, and very-low-birth-weight infants have less developed lungs and respiratory systems [20, 21]. Hypoxia-inducible factor-1α (HIF-1α) is one of the most important cytokines secreted by hypoxic cells. HIF-1α promotes angiogenesis and adapts the body for hypoxia by regulating downstream target genes, such as vascular endothelial growth factor. This process promotes the occurrence and development of IH. A three-dimensional microtumor formation of IH had confirmed the correlation between hypoxia and IH [22]. Therefore, preterm twins are more likely to develop morbidities. Further, lower-birth-weight twins are more prone to hypoxia due to the competition between the twins for maternal nutrition and oxygen. Therefore, the lower birth-weight twin is typically affected by IH in twin sets.

As a widely recognized risk factor for IH, birth weight < 2500 g has been shown to increase the possibility of disease; for every 500 g decrease in birth weight, the risk of disease increases by 40%.

Low-birth-weight infants are susceptible to injury-related diseases due to poor development, and vascular injury-related mechanisms lead to the occurrence of IH [20, 23, 24]. However, in twins, the summed weight did not significantly correlate with morbidity. But the affected twin were more likely to have a lesser birth weight than the other one.This may be due to an increased demand for nutrition and oxygen supply associated with a larger weight. When the supply cannot be fully achieved, the dual effects of hypoxia and injury increase the probability of IH development.

The clinical features of IH in twins are largely similar to general signs [13, 14, 17]; however, the differences corroborate existing knowledge regarding the origin of IH and provide clues and inspiration for further research, clinical treatment exploration, and aiding early screening.

We admitted a 3-month-old female infant, who was part of an artificially conceived pigeon twin pair born prematurely at 33 weeks, with a left arm IH. However, her twin brother was asymptomatic. Their parents were cautioned on the risk of IH development in both twins and that the second twin required monitoring. Approximately 1 month later, her brother developed asymmetrical cheeks with no skin color changes. Ultrasonography and magnetic resonance imaging revealed the left cheek mass to be subcutaneous IH. Both twins recovered well after early treatment using propranolol [17].

As data regarding twins with IH is relatively scarce, this study collected data from two treatment centers and included only 83 cases; the sample size was limited. Moreover, data collection was limited by the subjectivity of the parents, especially regarding information such as whether assisted reproduction was used.

Further studies, including data from more patients, are underway to validate the findings of this study and understand the incidence of IH in twins.

## Conclusion

The clinical characteristics of infantile hemangioma in twins were consistent with their epidemiological characteristics. Female sex, preterm birth, less birth weight, and assisted reproduction increased the probability of morbidity in both twins. Such characteristics supported the theory injury-related mechanisms and hypoxia pathway lead to the occurrence of IH. Analysis of more characteristics of infantile hemangioma in twins may assist further research and clinical treatment.

## Data Availability

All data and materials used during the this study are available from the corresponding author on reasonable request.
